# Factors associated with radiographic progression and neurologic decline in patients with isolated traumatic subarachnoid hemorrhage

**DOI:** 10.1186/s41016-024-00377-0

**Published:** 2024-08-06

**Authors:** Kaushik Ravipati, Inamullah Khan, Wesley Chen, Ravi Nunna, Aaron Voshage, Sasidhar Karuparti, Ismail Ziu, Michael Ortiz

**Affiliations:** Department of Neurological Surgery, University of Missouri Hospital and Clinics, Columbia, MO USA

**Keywords:** Traumatic brain injury, Subarachnoid hemorrhage, Radiographic progression

## Abstract

**Background:**

Complicated mild traumatic brain injury (cmTBI) is a common neurosurgical disorder that consumes a significant amount of healthcare resources without a clearly established benefit. Best practices for the management of cmTBI regarding triage, hospital admission, and the necessity for repeat imaging are controversial. Our objective is to describe the rate of radiographic progression and neurologic decline for isolated traumatic subarachnoid hemorrhage (itSAH) patients admitted to the hospital. We hypothesized that only a minority of itSAH patients suffer radiographic progression and that radiographic progression is not necessarily associated with neurologic decline.

**Methods:**

Database queries and direct patient chart reviews were used to gather patient data. *T*-tests and Fisher’s exact tests were performed.

**Results:**

A total of 340 patients with cmTBI associated with itSAH were included for analysis. The radiographic progression rate was 5.6%. There was no statistically significant association between age, gender, GCS at presentation, anticoagulation status, and risk of radiographic progression. However, subgroup analysis on anticoagulated patients did show those on warfarin had a statistically significant risk of radiographic progression (*p* = 0.003). No patient developed neurologic decline, irrespective of whether they developed radiographic progression.

**Conclusion:**

Secondary triaging, hospital admission, ICU stay, and repeat HCT might not be necessary for awake, GCS 13–15 patients with itSAH without any other significant injuries. In the case of anticoagulant use, but not necessarily antiplatelet use, the medication should be reversed, and admission should be considered.

## Background

Complicated mild traumatic brain injury (cmTBI) is defined as a Glasgow Coma Scale (GCS) score of 13–15 and head computed tomography (HCT) with evidence of intracranial injury, which may include skull fractures, a variety of intracranial hemorrhages as well as intracranial edema [1]. Traditionally, patients with cmTBI are admitted to the hospital for observation with serial imaging and/or intensive care unit (ICU) admission [2–6], although this has been highly variable given the lack of a definitive treatment algorithm.

As a consequence of the recent conscientiousness created by the continued limitation of healthcare resources in the United States, many groups have raised concerns related to the secondary over-triage of cmTBI [7–10] as well as the questionable necessity of repeat imaging in neurologically stable patients, along with its associated cost increase and radiation exposure [2, 11–13]. Particularly given that the overwhelming majority of these patients do not tend to decline and/or require neurosurgical intervention [4, 14–16].

A fundamental flaw that plagues most neurological trauma studies is the significant difficulty in isolating a specific pathology given the heterogeneity of the trauma population. Therefore, focus has been placed on identifying subsets of cmTBI that can be simplified in a way as to provide reproducible outcomes and findings. Isolated traumatic subarachnoid hemorrhage (itSAH) has been identified as a potential target subset and multiple studies have argued that ICU admission and/or repeat imaging might not be necessary [17–21].

The primary focus of this study is to describe the rate of radiographic progression in patients with itSAH as well as its relationship with neurologic decline. The secondary focus is to identify which patient variables are associated with neurologic decline and radiographic progression and to assess the necessity for repeat imaging and ICU admission. We hypothesized that only a minority of itSAH patients suffer radiographic progression and that radiographic progression may not be necessarily associated with neurologic decline.

## Methods

### Study design

This retrospective single-center study was carried out in a tertiary-care hospital, was approved by the institutional review board (#2012571), and was conducted in compliance with Health Insurance Portability and Accountability Act (HIPAA) regulations. Patient consent was waived by the institutional review board because of the minimal risk as a retrospective chart review study.

The neurological surgery patient census was queried to identify all patients with SAH treated in our institution from January 2014 to December 2021. Patients who met the following criteria were included: (1) age ≥ 18 years, (2) GCS 13–15 at presentation, (3) blunt mechanism of head trauma, and (4) itSAH without additional intracranial imaging findings. Patients who had any significant extracranial injuries were excluded. A significant extracranial injury was defined as an extracranial injury that would require admission for management, irrespective of whether it would require medical and/or surgical treatment. All the itSAH which were identified would be classified as modified Fischer grade 1 hemorrhages.

### Outcome measures

The primary outcome was to identify the rate of radiographic progression after diagnosis of cmTBI associated with itSAH. Radiographic progression was defined as the identification of an enlarging hemorrhage or new focus of hemorrhage on repeat CT scans of the head. Secondary outcomes included the rate of neurologic decline, measurement of the association between radiographic progression and neurologic decline, admission length of stay, disposition at discharge, and condition at the latest follow-up.

### Statistical methods

Descriptive statistics were performed. Categorical variables were summarized as *n* (%). Continuous variables were summarized as mean ± 1 standard deviation. Statistical analysis was performed on SPSS (IBM, Armonk, NY, USA). Independent *t*-tests were used to compare means for continuous variables. Categorical variables were compared with Fisher’s exact test or chi-square. Statistical significance in individual variables was determined by *p* < 0.05.

## Results

### Study cohort and patient characteristics

**Fig. 1:**
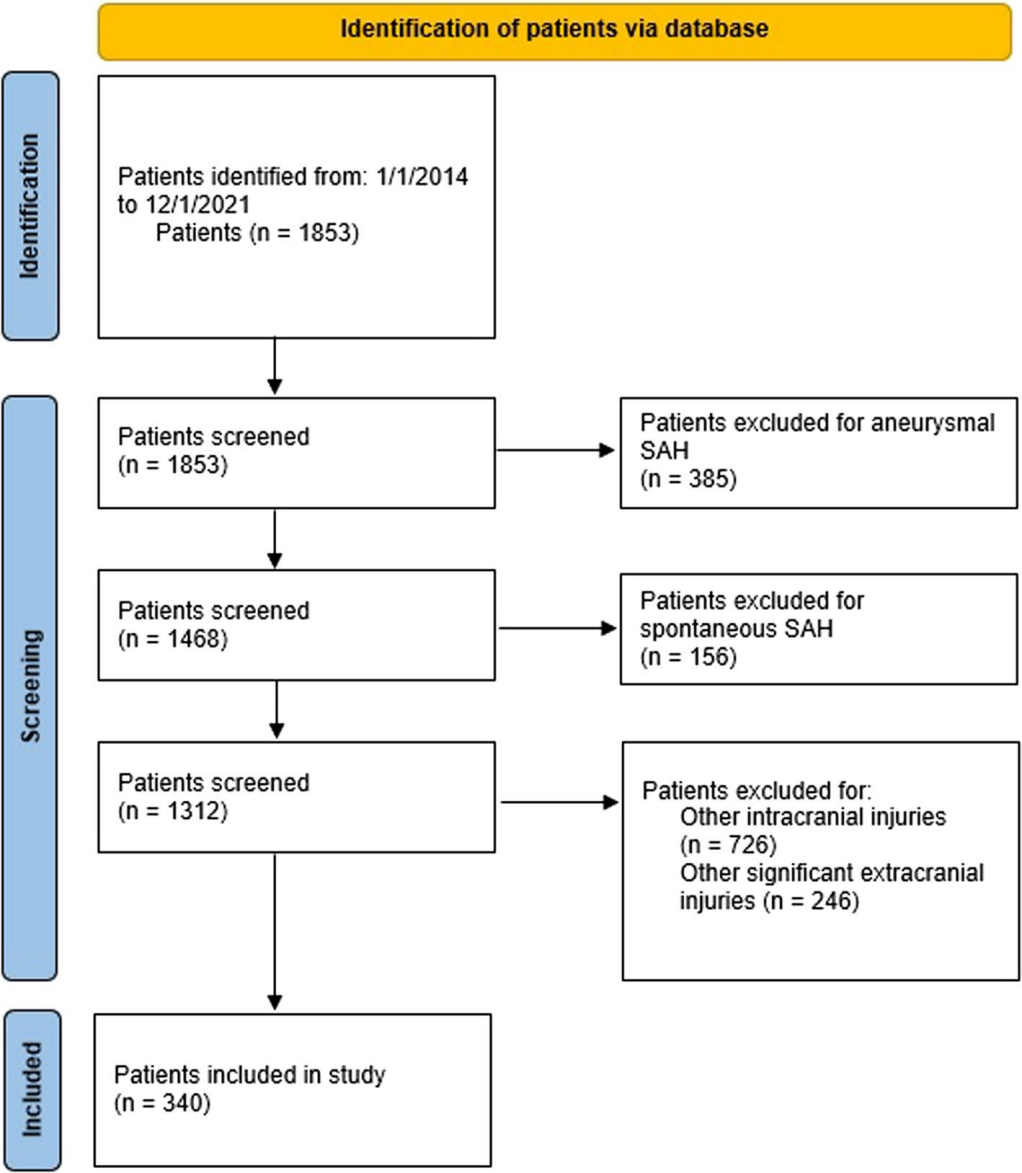
PRISMA

A total of 1853 patients were initially identified. After the application of inclusion and exclusion criteria, 340 patients were included for analysis (Fig. 1). Table 1 presents our population demographics and characteristics. The mean age at presentation was 61.95 ± 21.23. Male gender comprised 49.1% of the sample size. Eighty-seven percent of patients had a GCS of 15 at presentation. Coagulation status is presented in Table 2. Fifty-one percent of patients were taking anticoagulants, while 37.6% of patients were taking antiplatelets. The type of anticoagulants included vitamin K antagonists and non-vitamin K antagonist oral anticoagulants, while the type of antiplatelets included COX inhibitors as well as P2Y12 receptor inhibitors. All anticoagulants were reversed. Patients on anticoagulants plus antiplatelets had both reversed. Patients on antiplatelets alone were not reversed (Table 3).

**Table 1:** Population demographics

Variable	Total	No radiographic progression	Radiographic progression	*p* value
Sample (*N*)	340	321	19	–
Age (years)	62 ± 21.2	61.5 ± 21.1	69.3 ± 23.4	0.122
Gender				
Female	173 (50.9%)	165 (51.4%)	8 (42.1%)	0.43
Male	167 (49.1%)	156 (48.6%)	11 (57.9%)	1
GCS				
15	294 (87%)	280 (87.8%)	14 (73.7%)	0.076
14	39 (11.5%)	35 (11%)	4 (21%)	0.186
13	5 (1.5%)	4 (1.3%)	1 (5.3%)	0.1673

**Table 2:** Coagulation status

Variable	Total	No Radiographic progression	Radiographic progression	*p* value
Sample (*N*)	340	321	19	–
Anticoagulation/ Antiplatelet use	150 (44%)	139 (43.3%)	11 (58%)	0.239
Anticoagulation	51 (15%)	46 (14.3%)	5 (26.3%)	0.18
Warfarin	28 (54.9%)	23 (50%)	5 (100%)	**0.003**
Xarelto	10 (19.6%)	10 (21.7%)	0	–
Eliquis	6 (11.8%)	6 (13%)	0	–
Pradaxa	4 (7.8%)	4 (8.7%)	0	–
Others	3 (5.9%)	3 (6.5%)	0	–
Antiplatelets	128 (37.6%)	118 (36.8%)	10 (52.6%)	0.165
Aspirin	102 (79.7%)	94 (79.7%)	8 (80%)	0.982
Plavix	8 (6.3%)	8 (6.8%)	0	–
Aspirin/Plavix	14 (10.9%)	12 (10.2%)	2 (20%)	0.3428
Others	4 (3.1%)	4 (3.4%)	0	–
Coagulation studies (at presentation)				
INR	1.15 ± 0.5	1.14 ± 0.5	1.32 ± 0.5	0.107
PTT	29.7 ± 5.8	29.6 ± 5.7	31 ± 7.5	0.405
Platelet count (thousands)	219.8 ± 68.1	221.1 ± 67.9	199.3 ± 70.9	0.175
TEG inhibition				
AA (%)	17.1 ± 33	16.9 ± 32.8	20.8 ± 38.6	0.741
ADP (%)	17 ± 28.9	16.7 ± 28.5	21.3 ± 39.5	0.664
Medication reversed	33 (22%)	28 (20.1%)	5 (45.5%)	0.051

**Table 3:** Admission, disposition, and return to hospital

Variable	Total	No radiographic progression	Radiographic progression	*p* value
Sample (*N*)	340	321	19	–
Admission setting				
Floor	276 (81.2%)	263 (81.9%)	13 (68.4%)	0.144
ICU	57 (13.8%)	52 (16.2%)	5 (26.3%)	0.25
ER observation unit	7 (2.1%)	6 (1.9%)	1 (5.3%)	0.31
Length of stay (days)				
ICU LOS	0.5 ± 1.6	0.5 ± 1.6	0.6 ± 1.3	0.841
Floor LOS	2.6 ± 3.1	2.5 ± 3.2	3 ± 2.3	0.539
Total LOS	3.1 ± 3.3	3 ± 3.3	3.6 ± 2.2	0.489
Disposition				
Home	284 (83.4%)	270 (84%)	14 (73.7%)	0.24
Facility	56 (16.6%)	51 (16%)	5 (26.3%)	0.24
30-day ER revisit	13 (3.8%)	13 (4%)	0	-
30-day readmission	12 (3.5%)	12 (3.7%)	0	-

### Radiographic progression and neurologic decline

Nineteen (5.6%) patients had radiographic progression. Eleven (58%) of these were anticoagulated. There was no statistically significant association between age, gender, GCS at presentation, anticoagulation status**,** antiplatelet used, coagulation studies, platelet count or thromboelastography inhibition, and risk of radiographic progression**.** Although anticoagulation itself was not associated with an increased risk of radiographic progression, subgroup analysis on anticoagulated patients did show that a significantly higher proportion of patients on warfarin developed radiographic progression (*p* = 0.003) (Table 2). All patients with radiographic progression were identified during the first 24 h by repeat HCT. At the time of radiographic progression, no patient showed a decrease in their admission GCS. No patient had a neurologic decline, irrespective of whether they had radiographic progression.

### Hospital stay, disposition at discharge, and readmissions

Most patients were admitted to the floor (81.2%), and this varied depending on attending provider preference and patient comorbidities. The average length of stay (LOS) was 3.1 ± 3.3 days. Most patients were discharged home (83.4%). Patients who were discharged to a facility were older patients for which there was concern for fall risk. Aspirin was restarted 3 days after a stable scan and all other anticoagulants, including Plavix, were restarted 7 days after a stable scan. All patients (*n* = 13) who returned to the hospital within 30 days post-discharge were patients who had not had radiographic progression during their admission. Only 2 ER visits were related to the initial traumatic injury. The first one was for refractory headache and did not require admission. The second one was for new-onset seizures and required readmission for observation. Not all of these patients had repeat HCTs, but those who had did not reveal late radiographic progression. All patients were followed up within 6 weeks in the Neurosurgery Trauma Clinic and were found back at their baseline.

## Discussion

Complicated mild traumatic brain injury (cmTBI) is a common neurosurgical disorder that consumes a significant amount of healthcare resources without a clearly established benefit. In our study population, no patient had any neurologic decline during their hospital stay, irrespective of whether they had radiographic progression, and radiographical progression did not change hospital course. Additionally, radiographical progression did not influence return visits to the ER or exam findings 6 weeks later when followed in the clinic. These findings demonstrate the unclear benefit associated with repeat imaging, hospital admission, or neurosurgical consultation in itSAH.

### Neurosurgical triage, evaluation, and management of patients with cmTBI and itSAH

The typical triage pattern for cmTBI associated with itSAH is variable, but most institutions follow a similar algorithm. If the patient presents to a smaller hospital, they are then transferred to a tertiary hospital with neurosurgical services. The patients are then either admitted to an ICU or regular floor bed, another CT is obtained within 24 h, and the patient is subsequently discharged if they do not have any neurologic decline. This process has conventionally been repeated on every itSAH patient under the presumption that there is a small but real possibility that any of these injuries can worsen and potentially require intervention. However, evidence from our institution and from previous studies performed at other institutions indicate that appropriate patient selection can potentially absolve the need for current practice patterns.

Multiple studies have investigated the need for current management standards regarding traumatic subarachnoid hemorrhage in mild TBI with a specific focus on whether neurosurgical consultation, ICU stay, or repeat brain imaging is required. Studies have found that repeating an HCT is not required if the patient does not have any neurologic decline [22]. Additionally, selectivity in ordering repeat HCT in mild TBI has led to decreased hospital LOS without any negative impact on GCS and patient outcomes [4]. A similar study to ours is in concordance with these findings as it reported that patients with mTBI present a low rate of neurologic decline and, even when they did have neurologic decline, there was resolution without any significant intervention and no significant benefit to ICU stays [21].

Given that studies appear to lean towards low utility in repeating CT imaging as well as no significant benefit in ICU stay for this patient population, the next consideration would be whether a neurosurgical consult is required for this specific group of patients. Traditionally any traumatic intracranial hemorrhage, irrespective of significance, has warranted neurosurgical consultation. However, current studies indicate that this may not be necessary for the itSAH population [23]. It has been argued that even transfer to a tertiary center with neurosurgical capabilities could potentially be avoided in this population [24].

One of the major benefits of reducing interfacility transfer, reducing repeat imaging, and reducing admissions for this specific patient population is reducing unnecessary resource consumption. Kuhn et al. indicated that at one hospital, just the transportation costs of potentially avoidable transfers were $1.46 million over 2 years [25]. Diagnostic, treatment, and admission costs vary between institutions but are anything but nominal for this patient population. At our institution, the quoted total cost including the evaluation of a plain HCT is ~ $800. Inpatient admission to the floor is approximately $1800 a night, while ICU admission costs $7500 a night. This does not include any diagnostic work-up (imaging, labs, etc.) or necessary treatments. As a rough example, taking our data on LOS and assuming admission only to the floor, an inpatient admission would equate to approximately up to $11,520. Not including the initial or repeat HCT which would sum the cost up to approximately an additional $1600. Additionally, removing the repeat HCT from the algorithm would reduce radiation exposure. Although an HCT only has an estimated dose of 2 mSv, the carcinogenic risks of accumulated exposure to ionizing radiation are well described [26]. Reducing admissions for these patients would decrease the overall toll on hospital occupancy in tertiary centers, and free up beds for patients who truly require the resources of a tertiary center. Additionally, avoiding unnecessary transfers would also reduce the emotional toll on patients and their families, as in our institutional experience, they are often moved hundreds of miles away from their homes and their support resources.

### Transitioning into a clinically and cost-effective algorithm

As resources vary in each tertiary hospital system as well as their surrounding local facilities, we propose a graduated progression toward a new triage protocol. The first step would be to discontinue repeat CT imaging in the itSAH population. There is evidence to support that in patients with intracranial hemorrhage with GCS at presentation > 12, with no coagulopathies, and return to GCS of 15 at 24 h, repeat HCT did not change management or predict the need for intervention [27]. Our study is in agreement with and further supports that the selection of patients for only itSAH with GCS > 12 may have reduced the need for repeat CT imaging even further, as coagulopathy did not predict progression on imaging, and progression on imaging did not predict the need for intervention as no one in this patient population required intervention.

The second step would be to discontinue inpatient admission for these patients. It has been suggested that observation in an ED unit for 24 h can be just as effective as inpatient admission for mild TBI patients and may lead lower rate of neurosurgical consultation and repeat HCT compared to the equivalent inpatient group, in addition to a reduced length of stay [28]. However, this conclusion should only initially be generalized to large academic centers where neurosurgery is available and ED staff is trained appropriately for neurological exams, so neurologic decline can be effectively recognized in a timely fashion.

The last, and likely most difficult step, would be to avoid transferring this patient population to tertiary centers altogether. The difficulty with taking this last step would be ensuring appropriate patient selection by transferring hospitals, as well as confidence on the part of transferring hospital physicians that they are appropriately treating the patient. One way to ameliorate these insecurities would be to have a neurosurgeon evaluate each case in real-time via teleconsultation. In a pilot study by Alan et al. [29], they trialed having a neurosurgeon evaluate imaging and neurological exams via teleconsultation for TBI patients with GCS 14–15 and abnormal CT head to be considered for transfer. Compared to the control group in which all patients were transferred, none of the patients who were screened and not transferred had any deterioration. This would significantly reduce costs related to transferring all patients in this group automatically to tertiary centers and would also reduce the significant burden placed on these patients’ families from displacement.

The goal outcome would be that cmTBI patient care can be protocolized so that emergency room physicians in non-tertiary hospitals would feel comfortable selecting this patient population and observing them without transferring to a tertiary center and consuming unnecessary resources. This would in turn lead to a change in practice patterns so as to decrease unnecessary hospital admissions, hospital LOS, and unnecessary repeat HCTs and decrease overall cost without sacrificing clinical efficacy or compromising patient safety. Our study continues to add to the increasing body of literature that supports this and, furthermore, identifies a specific low-risk patient population that may represent an excellent pilot population for the implementation of such protocols.

### Limitations

This study is a retrospective single-center experience and, as such, carries all the inherent bias of retrospective data collection, as well as the bias of individual surgeon management preferences. Additionally, we present a relatively small sample size. However, this is a highly specific and generalizable group of patients which may ameliorate the sample bias effect. There is no consensus as to how much SAH constitutes “too much” SAH. We do not specify the amount of hemorrhage. SAH volume analysis and establishment of definitions might be the next step moving forward. In addition, significant variability and external factors such as comorbid medical conditions, concurrent alcohol and drug use, and geography may influence triage and management patterns based on regional distribution.

## Conclusions

We identified a specific subset of patients with cmTBI associated with itSAH and found they are at a low risk of radiographic progression and at an even lower risk of neurologic decline. Our population’s risk of radiographic progression was 5.6%, and none of those patients had neurologic decline. Anticoagulation status, when appropriately reversed, did not specifically predict radiographical progression, except in the group of patients anticoagulated with warfarin. None of the patients in our study required neurosurgical intervention. Our findings suggest that repeat HCT, inpatient admission, and potentially even transfer to a tertiary center might not be necessary for this specific group of patients.

## Data Availability

The datasets used and/or analyzed during the current study are available from the corresponding author on reasonable request.
